# Foreign Body Granuloma and Infection‐Related Complications Arising From Acupoint Embedding: A Case Study and Literature Review

**DOI:** 10.1111/jocd.70718

**Published:** 2026-02-09

**Authors:** Xiazhen Xu, Kaihua Song, Yang Shen, Chenxin Jang, Shouhai Hong

**Affiliations:** ^1^ Zhejiang Chinese Medicine University Hangzhou Zhejiang China; ^2^ Department of Acupuncture and Moxibustion, the First Affiliated Hospital of Zhejiang Chinese Medical University Hangzhou Zhejiang China

**Keywords:** acupoint embedding (AEM) therapy, complications, cosmetic, foreign body granuloma, lose weight, nontuberculous mycobacteria

## Abstract

**Background:**

Acupoint embedding (AEM) therapy merges traditional acupuncture with modern biomaterials to treat various diseases and for cosmetic uses, especially obesity and facial anti‐aging.

**Research Objective:**

To examine complications from acupuncture embedding therapy (AEM), focusing on foreign body granulomas and infections. The study reviews cases to understand their causes, symptoms, diagnosis, treatment, and prevention, aiming to offer new treatment options for granulomas from acupuncture.

**Methods:**

Using PRISMA guidelines, a literature search was conducted in PubMed, Embase, Web of Science, and the Cochrane Library with keywords related to thread embedding acupuncture and its adverse effects, covering 2014–2024. Nine articles were included, along with a 2023 case report of a woman developing a granuloma after collagen thread acupuncture for weight loss.

**Results:**

Among the 11 cases of complications following acupuncture point thread implantation, foreign body granuloma accounted for 90.91%, making it the most prevalent type of complication associated with this therapy. This case report details a treatment involving antibiotics to reduce exudate, steroids to shrink the mass, and later, a combination of warm acupuncture and radiation therapy to further reduce the nodule. However, surgical treatment yields the most significant therapeutic effect for foreign body granulomas.

**Conclusion:**

Animal‐derived sutures, like catgut, often cause foreign body granulomas quickly. If these granulomas resist treatments like steroids or antibiotics, surgery is the best option. Clinicians must recognize the high risk of granulomas and prioritize using biocompatible sutures, maintaining aseptic techniques, ensuring precise suture placement, and closely monitoring patients post‐surgery. For small early‐stage nodules, consider medication and warm acupuncture. For stubborn or larger lesions, prompt surgery is vital to reduce complications and improve outcomes.

## 1 Introduction

Acupoint embedding (AEM) therapy represents an innovative approach that integrates traditional acupuncture with contemporary biomaterials. This therapeutic modality involves the insertion of intestinal or surgical threads into specific acupoints to achieve sustained stimulation [1, 2]. AEM technology advances acupuncture therapy by leveraging the principles of traditional Chinese medicine to maintain continuous acupoint activation. Unlike conventional acupuncture, where needles are removed after a short period, the threads in AEM remain embedded within the tissue for an extended duration, thereby providing ongoing mechanical stimulation to the acupoints and adjacent regions [3]. Acupuncture, recognized as a form of green therapy, has disseminated from Oriental countries to Europe and the United States since the 16th century [4]. In the 1960s, Chinese acupuncturists discovered that embedding threads at acupoints could extend stimulation and decrease treatment duration. Prior to 2010, reports of complications associated with AEM were scarce, particularly in international contexts. However, post‐2010, there has been a notable increase in related research, and the safety of AEM has garnered significant attention globally [5]. A recently published Korean multicenter, prospective, observational study systematically evaluated the safety of thread embedding acupuncture (TEA) therapy in a real‐world clinical setting across multiple centers for the first time [6]. AEM is utilized in the treatment of various medical conditions, including osteoarticular and soft tissue diseases [7, 8, 9, 10, 11, 12], neurological disorders [13, 14], allergic rhinitis [15], obesity and metabolic syndrome [16, 17, 18, 19, 20, 21], diabetes [22], gynecological conditions [23, 24, 25], and gastroesophageal reflux disease [26]. Additionally, AEM is employed in facial aesthetics to diminish wrinkles and improve skin elasticity [27, 28, 29]. AEM, as a procedure involving the implantation of foreign bodies, can potentially elicit a range of uncommon foreign body reactions (FBR) during the intended stimulation of acupoints. Common manifestations include induration, bleeding, ecchymosis, redness, swelling, and pain. Relatively rare FBRs are foreign body granuloma and infection‐related complications [5]. The inflammatory response triggered by exogenous substances and foreign bodies implanted in the skin constitutes a FBR, which may lead to a granulomatous response in the skin. Foreign body granuloma (FBG) represents the primary manifestation of chronic FBR and is classified as a grade 4 FBR [30]. The FBR classification, as delineated by Duranti et al., elucidates the histopathological characteristics of FBG, which include capsule implants and significant foreign body reactions [31], Granuloma represents a chronic inflammatory response with diverse etiologies and can be defined as a tumor‐like formation composed predominantly of immune cells, particularly macrophages [32]. Macrophages are the principal cellular components of granulomas. In FBG, these macrophages become activated and coalesce to form multinucleated giant cells [33]. These giant cells are characterized by the random arrangement of more than 20 nuclei [32]. Activated macrophages are occasionally referred to as epithelioid cells due to their morphological resemblance to epithelial cells [34]. Typically, FBG associated with AEM presents as giant cell granuloma containing foreign body material [35, 36]. Additionally, infectious granulomas [37, 38], foreign body cystic granuloma, and abscess [39] may also be observed. The study identified that the primary factors contributing to complications, such as FBG and infections, are related to the implantation materials and clinical techniques employed. Initially, AEM utilized materials such as chromic sheep catgut threads and collagen threads. Early research indicated that the animal origin and enzymatic properties of sheep catgut threads were associated with a high incidence of adverse events (AEs), including nodules, cysts, and masses [36, 40]. Consequently, a search for alternative materials was undertaken. The development of biodegradable materials, such as polyglycolic acid (PGA), polylactic‐co‐glycolic acid (PLGA), polydioxanone (PDO), chitosan, and medicinal threads, has significantly reduced the rate of complications [40, 41, 42, 43, 44].

This case report details a patient who developed a delayed granuloma two weeks after collagen thread implantation at abdominal acupoints, resulting in localized induration, ulceration, and pain. Diagnosed as an FBG, the condition improved with antibiotics, corticosteroids, and warm acupuncture, but minor induration and pigmentation persisted a year later. The report underscores the persistent complications of thread implantation and reviews similar cases, aiming to discuss prevention and treatment strategies, especially regarding implant material selection, to encourage the use of new absorbable materials in facial aesthetics and abdominal fat reduction.

## 2 Case Report

On November 5, 2023, a 37‐year‐old woman visited our hospital after developing a granuloma reaction from acupuncture treatment for abdominal obesity at a local hospital. Antibiotics and steroids administered there were ineffective. She had previously received treatment involving AEM at a local hospital, which led to a granulomatous reaction. The local hospital administered antibiotics and hormone therapy, but these interventions proved ineffective. Consequently, she sought further treatment at our facility.

### 2.1 Initial Presentation and Prior History

The patient, a native of Zhejiang Province, had a history of childbirth and no prior diagnosis of metabolic syndrome. Clinically, she presented with multiple firm nodules in the abdominal region, accompanied by marked tenderness. One month before her clinic visit, she had undergone a single course of abdominal Acupoint Catgut Embedding (AEM) surgery for the treatment of abdominal obesity at a local hospital. In the abdominal region, acupoints Tianshu (ST25), Zhongwan (RN12), Guanyuan (RN4), Qihai (RN6), Xiawan (RN10), Daheng (SP15), Shuifen (RN9), Shuidao (ST28), Huaroumen (ST24), Wailing (ST26), Daimai (GB26), and Liangmen (ST21) were embedded with Shandong Boda Absorbable Surgical Suture (Collagen), size 3–0, length 2 cm, using the Acupoint Catgut Embedding technique. Five days post‐operation, mild abdominal swelling was noted without significant pain. By the tenth day, palpable abdominal nodes appeared with mild pain (VAS 2/10). After two weeks, multiple subcutaneous nodules formed at suture sites, causing persistent dull pain (VAS 7/10) and discharging a small amount of yellow fluid. On October 29, 2023, a local hospital evaluation showed no significant inflammatory markers. An abdominal ultrasound revealed several hypoechoic nodules about 1.2 cm from the subcutaneous layer, with the largest being 1.2 × 0.7 cm, having a well‐defined border and irregular shape. Despite initial treatment with oral antibiotics and hormone therapy, the patient continued to suffer significant pain from hard nodules, leading to a referral to our hospital. At the outpatient clinic, the patient had multiple hard abdominal nodules with localized tenderness and a VAS pain score of 5. There was a history of rupture and fluid discharge, but no erythema, swelling, fever, or redness. The patient's personal and medical history was unremarkable, with no prior skin conditions, allergies, or immune disorders.

### 2.2 Physical Examination

**FIGURE 1:**
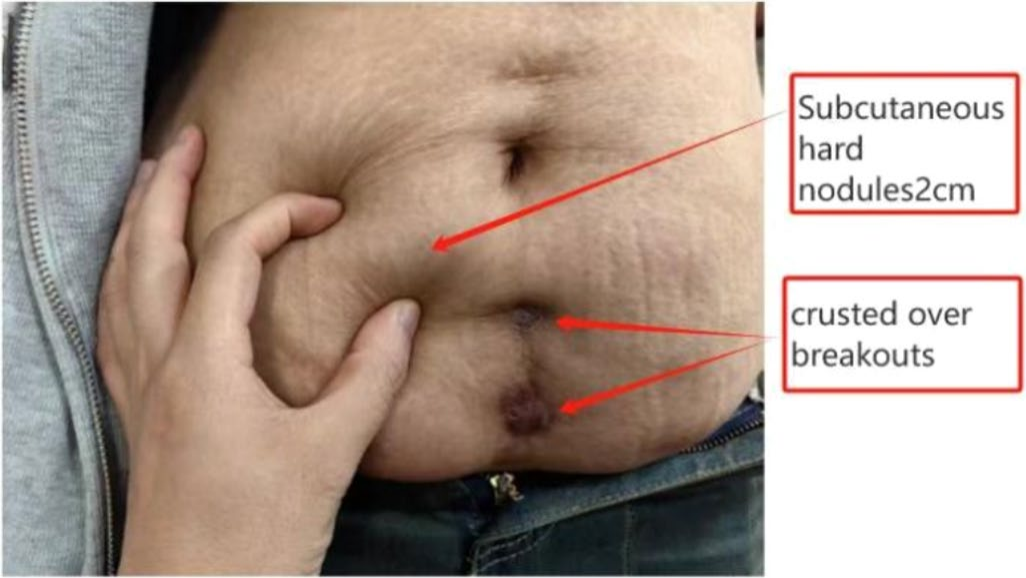
Multiple subcutaneous nodules about 2 cm in size in a linear distribution on the abdomen, with crusted ulcers.

The patient weighs 65 kg and is 161 cm tall. They have 19 hard subcutaneous nodules, each about 2 cm by 2 cm, in the abdominal area, causing moderate tenderness. No rash, skin discoloration, fluctuation, or drainage is present. Coin‐sized broken scabs with residual scarring are observed at the Qihai and Guanyuan acupoints. (As shown in Figure 1).

### 2.3 Treatment

Based on the patient's clinical symptoms and relevant auxiliary examinations, a diagnosis of FBG was established. The patient was administered a single injection of 1 mL of betamethasone compound injection into the buttocks for its anti‐inflammatory effects. Concurrently, a local “circumferential stabbing method” was employed, involving the insertion of four needles at a 45° angle around the periphery of the nodule. Additionally, a needle was inserted perpendicularly into the center of the nodule using 0.30 × 40 mm needles, with one needle penetrating to the base of the nodule. A moxa pillar was plAEMd at the center of the needle handle to facilitate warm needle acupuncture. Furthermore, TDP irradiation was applied, covering the entire affected area, with the duration synchronized with the needle retention time of 30 min.

### 2.4 Methods of Literature Review

An extensive literature review was conducted by searching databases such as PubMed, Embase, Web of Science, and the Cochrane Library using keywords such as “Thread Embedding Acupuncture,” “Catgut Embedding,” “thread embedding,” and “acupoint embedding.” This search aimed to collate information regarding adverse reactions, adverse events, side effects, complications, risks, and safety associated with these procedures. The search covered the period from 2010 to 2025. From the retrieved articles, case reports published within the last 15 years were selected for inclusion in the study (as detailed in Table 1). Ultimately, nine case reports detailing complications of catgut embedding, involving a total of 12 patients, were identified.

**TABLE 1:** The characteristics of the 10 cases.

Years	Author	Organization	Age/Genders	Purpose of ACE	Underlying disease/allergy history	Parts of ACE	Materials for ACE	Key clinical presentations	Types of complications	Treatment	Outcomes	Source (journal abbreviation)
2011	Chuang et al.	Department of Dermatology, China Medical University Hospital	27‐year‐old/woman	Loose weight	None	The lower abdomen and both medial thighs	Catgut embedding treatment	Multiple, tender, bean‐sized erythematous nodules with central darkened points in a linear arrangement over both medial thighs and over the lower abdomen after 1 month	Foreign body granuloma	Partial excisional biopsy of nodes with intra‐lesional steroid injection	Nodules regressed spontaneously with postinflammatory hyperpigmentation after 6 months	*Acupunct Med*
2014	Noh et al.	Department of Dermatology, Asan Medical Center, University of Ulsan College of Medicine	44‐year‐old/woman	Cosmetic	None	Lower aspects of both cheeks and nasolabial folds	polydioxanone sutures	Multiple tender erythematous papules and nodules on both cheeks after 1 month	*Mycobacterium fortuitum* infection	combination therapy with oral doxycycline, ciprofloxacin, and clarithromycin.	The lesions continued to improve after 3 months and a 6‐ to 12‐month course of combination therapy was recommended	*J Am Acad Dermatol*
2015	Beigi et al.	Department of Vascular Surgery, Alzahra Hospital, Isfahan University of Medical Sciences	27‐year‐old/woman	Treating Diseases	Inflammatory bowel disease	Bilateral lower limbs	Catgut embedding treatment	Unilateral soreness and non‐pitting edema of the right lower extremity	Cold abscess	Ineffective broad‐spectrum antibiotic therapy, surgical excision of abscess and removal of sutures	The leg oedema had resolved by 2 months after surgery	*Acupunct Med*
2020	Li et al.	Department of Dermatology, Peking University First Hospital	57‐year old/woman	Loose weight	None	Abdomen and waist	Catgut embedding treatment	Indurated erythema on her abdomen and waist. Multiple subcutaneous hard nodules were palpated, ranging from bean‐sized to egg‐sized, with poor mobility, and skin was felt warm.	Foreign body granuloma	Betamethasone intralesional injection and topical corticosteroid without significant improvement.	Not resolve after 9 months	*Ann Dermatol*
2021	Wang et al.	Department of Dermatology, Department of Aesthetic Medicine and Drug Hypersensitivity Clinical and Research Center, Chang Gung Memorial	44‐year‐old/woman	Loose weight	None	Abdomen, flanks and thighs	Catgut embedding treatment	Multiple pruritic, enlarging tumors with discharge aligned in a linear pattern on the abdomen, flanks and thighs	Foreign body cystic granuloma and abscess	The lesions were removed by surgery	The patient's pruritus was markedly reduced	*Br J Dermatol*
2021	Zhou et al.	Department of Ophthalmology, Jilin University Second Hospital	13‐year‐old/boy	Myopia treatment	None	The bilateral periocular acupoints	Catgut embedding treatment	Pain in his right eye, followed by reduced vision, With the intraocular foreign body (catgut) residue.	Anterior uveitis + intra‐ocular foreign bodies	Topical corticosteroids, cycloplegics, and topical and intravenous antibiotics were administered. a diagnostic vitrectomy was performed for further treatment.	At 3 months postoperatively, the patient seemed stable, with irreversible retinal damage.	*Clin Exp Optometry*
2022	Yook et al.	Department of Dermatology, Seoul St. Mary's Hospital, College of Medicine	50‐year‐old/woman	Cosmetic	None	Face	Gold thread	Asymptomatic erythematous induration	Foreign body granuloma	Multiple intralesional corticosteroid injections, oral prednisolone + suture removal	Lesions showed slight improvement six months later but remained.	*Indian J Dermatol Venereol Leprol*
2024	Xing et al.	Department of Laboratory Medicine Daping Hospital Army Medical Center of the PLA	36/51/40 year‐old/woman	Loose weight	None	Waist and abdomen, bilateral hip joint	Silk protein	Several subcutaneous masses, Swelling, tenderness, fluctuation and ulceration, Excreting yellow white purulent secretions	Non‐tuberculosis Mycobacterium infection	Three antibiotics, cefmetazole sodium, amikacin, and clarithromycin, combined with 5‐aminolevulinic acid photodynamic therapy (ALA‐PDT)	After 3–6 months of treatment, all patients' skin lesions subsided	*Clin Lab*

### 2.5 Results From the Literature Review

The study conducted a review of 11 cases involving complications arising from AEM, inclusive of the present case. The predominant complication identified was FBG, which constituted 90.91% (10 cases) of the total, while simple infection complications were observed in only one case (9.09%). Among the 10 patients with FBG, 80.00% (8 cases) were female, with an average age of 37.6 years, spanning from 13 to 57 years. Regarding the suture materials used, catgut sutures were the most prevalent (55.00%, 6 cases), followed by collagen sutures (9.00%, 1 case), polydioxanone (PDO) sutures (9.00%, 1 case), and silk protein sutures (27.00%, 3 cases). Of the 11 cases, 70% exhibited normal inflammatory markers, with no fever or leukocytosis. Three cases of non‐tuberculous mycobacterial infections were confirmed through positive pus cultures and acid‐fast staining. The detection period for FBG varied from 1 week to 6 months, with an average latency period of 6.5 months. Clinically, all 10 patients presented with subcutaneous nodules (100% incidence), 9 cases (90.00%) exhibited swelling and tenderness, and 6 cases (60.00%) experienced purulent discharge. High‐incidence sites were concentrated in the abdomen (6 cases, 60.00% for weight loss purposes) and face (4 cases, 40.00% for cosmetic purposes). Regarding treatment regimens, five cases (45.00%) received intralesional steroid injections, while seven cases (70.00%) underwent antibiotic therapy due to concurrent infections or inflammatory responses. Surgical intervention proved most effective for foreign body granuloma, with 6 cases (60.00%) undergoing surgical management.

A comprehensive analysis of the selected articles was undertaken to synthesize the complications linked to AEM weight loss and cosmetic treatments. The characteristics of the 10 cases are presented in Table 1.

## 3 Discussion

FBG represents a chronic proliferative inflammatory response of the body to exogenous or endogenous substances that are non‐degradable or non‐eliminable [45]. This response represents the body's attempt to eliminate the foreign material. Histologically, granulomas are composed of an inflammatory infiltrate consisting of histiocytes and epithelioid cells. They are distinguished primarily by the proportion and arrangement of lymphocytes, plasma cells, neutrophils, eosinophils, and multinucleated giant cells, as well as the presence of polymorphous exudates and, occasionally, necrosis [46]. Graivier et al. have classified FBG into non‐inflammatory and inflammatory types, with each category further subdivided into early and late stages [47]. Early granulomas develop through various pathological processes, including epidermal fibrosis leading to hypertrophic scars, irregular skin reactions, allergic responses causing capillary dilation, immune hypersensitivity reactions forming lumps or nodules, and inflammatory reactions resulting in abscesses [46, 48]. Late‐stage granulomas from dermal fillers appear in three forms: cystic granulomas from collagen and hyaluronic acid, potentially leading to sterile abscesses; edematous granulomas from permanent fillers like silicone, causing swelling and inflammation; and sclerotic granulomas from granular injectables such as Artecoll, typically developing 6 to 24 months post‐injection. Occasionally, granulomas may exhibit mixed characteristics [49]. Since 2006, routine histopathological examinations have been discontinued once a clinical diagnosis is confirmed through a questionnaire [50]. Biofilm‐associated FBG represents a delayed‐onset condition wherein implants placed in the skin or subcutaneous tissue may develop biofilms. The most effective strategy for preventing biofilm formation involves cleansing the injection site with preparations of povidone‐iodine, chlorhexidine, or benzalkonium chloride prior to injection. The initial approach to treating patients suspected of having biofilm‐associated infections involves combination therapy with broad‐spectrum antibiotics, typically including quinolones and macrolides [47], These cases of FBG infection caused by Mycobacterium *Mycobacterium fortuitum* and non‐tuberculous mycobacteria following AEM, necessitating combination multiple antibiotics, exemplifies a biofilm‐associated infection [38, 51]. Evidence suggests that the dermis exhibits higher immunoreactivity compared to subcutaneous or dermal‐subcutaneous junction administration, which leads to an increased propensity for granuloma formation following intradermal injection [30, 46]. AEM filaments implanted in subcutaneous fat and muscle layers show a lower risk of FBG formation than dermal fillers, which can lead to foreign body giant cell granuloma. Histopathology shows varying tissue cell aggregates in the dermis with few lymphocytes and basophilic material in cells. Giant cell nuclei are diffusely distributed [35]. Some cases progress to Inflammatory FBGs [38, 39, 51, 52].

A review by Wang et al. [30] found most FBGs were treated with steroid injections (25/21.37%), surgical excision (18/15.38%), laser therapy (5/4.27%), and antibiotics (2/1.71%), with some using allopurinol, methotrexate, and fluorouracil. Notably, most refractory cases were predominantly addressed through surgical excision [49]. The indications for surgical intervention included: (1) a history of multiple unsuccessful non‐surgical treatments, (2) well‐defined and localized chronic lesions exceeding 3 cm in diameter, (3) severe signs of infection accompanied by sensory dysfunction or tissue necrosis, and (4) the emergence of psychological issues due to the recognition of significant contour deformity [50]. A review by Huang et al. suggests that granulomas induced by AEM may be closely associated with the characteristics of the thread, the standardization of the procedure, and the individual's immune status [5].

### 3.1 Granulomas and Infections Caused by Implanted Materials

A 37‐year‐old woman developed FBG after collagen thread implantation. These threads, similar to those from sheep intestines, require proteases for breakdown and absorption, which is difficult to regulate. Their animal origin increases the risk of tissue rejection, causing granulomas and infection [53]. Clinical cases by Wang et al., Furong Li et al., Yung‐Ting Chuang et al., and Beigi et al. have shown that embedding acupoint threads for weight loss can cause pruritic lumps with oozing, erythema, and nodules. These are histologically identified as cystic granulomas and foreign body abscesses. The sheep intestinal threads (Catgut) used can trigger an immune response, causing a delayed hypersensitivity reaction that results in granuloma formation [35, 45]. Additionally, the patient's body may exhibit rejection and allergic responses to this foreign protein, potentially causing symptoms such as elevated temperature, redness, rash, and itching at the site of thread embedding [53]. Prolonged inflammation may progress to abscess formation [39] or result in cold abscesses, which lack significant localized symptoms of redness or heat [52].

The study by Yook et al. on FBG formation linked to gold wire implants shows that repeated bending of the wire can release gold ions, like Au3+. These ions interact with lysosomal proteins, sensitizing T cells and triggering granulomatous inflammation. Despite gold's inert reputation, macrophage‐produced oxidants in the body can promote gold ion release, leading to a T‐cell‐dependent immune response [37, 54].

This paper analyzes FBGs and abscesses caused by catgut and gold threads, focusing on AEM‐induced granulomas. Key findings include: a history of AEM use, erythematous nodules that sometimes rupture and discharge pus, and histopathology showing histiocyte clusters with some lymphocytes and basophilic material in histiocytes. The patient's white blood cell count was normal. Antibiotics and hormones were ineffective, suggesting surgical thread removal as a potential solution. The case study highlights chronic granulomas from collagen threads, initially managed with antibiotics and hormones for aseptic inflammation. Subsequently, a combination of warm acupuncture and peri‐acupuncture was administered, leading to a reduction in granuloma size following conservative treatment. The author suggests that the mechanism may involve a dual effect: local thermotherapy may accelerate collagenase (MMP‐1) activity [55] by increasing the stability of the enzyme or altering its conformation, resulting in a shorter degradation cycle of the sheep's intestine threads; meanwhile, acupuncture reduces the release of IL‐1β by inhibiting the activation of NLRP3 inflammatory vesicles [56].

In acupoint embedded thread therapy, the choice of material is crucial for effectiveness and safety. The materials have progressed from natural options like Catgut and collagen to synthetic polymers such as PLA–ethanolic acid or polyoxazolone, and biologically active threads with features like antimicrobial agents, silver nanoparticles, or slow‐release capabilities [40, 57]. PGA has demonstrated efficacy in the management of simple obesity in adults [42]. Despite lacking flexibility, materials are improved via oxygen plasma and chitosan coating. For instance, Fu et al. reported that the modified PLGA exhibited a markedly reduced contact angle, significantly increased inhibition rate of *Staphylococcus aureus* , and enhanced fibroblast proliferation rate [43]. These modifications can substantially enhance material performance, augment therapeutic outcomes, and offer a novel direction for the optimization of future implantable wire materials.

### 3.2 Infectious Complications Related to Operating Techniques

In 2014, Noh et al. [38] reported a rare mycobacterial infection following acupoint thread embedding. A patient developed painful red facial nodules a month after PDO thread insertion. Tests confirmed a mycobacterial infection, which improved with oral antibiotics within 3 months. Similar non‐tuberculous mycobacterial (NTM) infections have been noted after tattooing and acupuncture. The authors suggested that the infection might have originated from skin‐colonizing mycobacteria, as disposable instruments were not sufficiently sterilized.

In 2019, Ahn et al. [58] documented a case of cellulitis attributed to the embedding of polydioxanone (PDO) wires, wherein the patient developed multiple tender nodules on the cheek three months post‐procedure. CT scans showed subcutaneous nodules, and histopathology revealed chronic granulomatous inflammation with abscesses. After surgically removing the PDO wires, tenderness quickly subsided, and swelling decreased within two weeks. The authors suggest that improper placement of the wires by non‐medical personnel caused repeated trauma, leading to this condition.

In 2021, Zhou et al. [59] reported a case of endophthalmitis caused by the improper implantation of periocular enteric wire, leading to pain and vision loss in the patient's right eye. A Doppler ultrasound and diagnostic vitrectomy revealed the wire had pierced the sclera and lodged in the retina, causing retinal detachment and endophthalmitis. Three months later, the patient's vision was reduced to light perception due to aseptic inflammation and retinal damage. The authors attributed the complication to the operator's lack of anatomical knowledge and poor control over needle insertion depth.

In 2024, Xing et al. [51] documented a *Mycobacterium abscessus* outbreak from enteric thread filament protein injection. Three patients developed erythematous, fluctuating masses after informal implantations. Pus cultures identified ST279, resolving in 3–6 months with cefmetazole, amikacin, clarithromycin, and photodynamic therapy. Contributing factors were device reuse, silk protein contamination, and poor conditions. NTM infection is a potential complication of acupoint thread therapy, caused by inadequate sterilization in informal settings and NTM's antibiotic resistance and biofilm formation, necessitating drug susceptibility‐guided treatments [60]. These biofilms have complex polysaccharide‐rich structures that host various microorganisms, helping them avoid the immune system and block macrophage phagocytosis. Studies show they often contribute to bacterial antibiotic resistance and tolerance [61, 62].

### 3.3 Complications Related to Individual Patient Differentiation Factors

The patient's underlying disease greatly affects the risk of AEM. Beigi et al. noted cold abscesses in a patient with inflammatory bowel disease after such a procedure. This disease causes prolonged immune activation in the intestines, leading to potential systemic immune issues and abnormal inflammatory reactions to goat intestinal threads, triggering a Th1 cell‐mediated response. The patient's immune dysfunctions, such as impaired phagocyte function and imbalanced regulation, disrupted the intestinal barrier and microbial balance, causing an overgrowth of pro‐inflammatory bacteria and a decrease in commensal bacteria, which activated the intestinal immune response and induced inflammation [63]. The patient exhibited no fever, maintained a normal white blood cell count, and had a negative abscess culture, indicating the formation of a cold abscess rather than a typical pyogenic infection.

## 4 Conclusion

While acupoint embedding threads have advanced to include materials with high biocompatibility with human tissue, the tolerability and long‐term safety of these materials in weight loss and cosmetic procedures remain areas of concern. This study examines a recent clinical case of FBG resulting from acupoint embedding thread therapy. In conjunction with documented cases of FBG and infection‐related complications from 2011 to 2024, this systematic review underscores that the safety of AEM therapy is largely contingent upon patient‐specific factors, the material of the thread, and the technique employed by the practitioner. The GB/T 21709.10–2008 standard [64] recommends prioritizing materials with superior biocompatibility. For FBGs measuring less than 3 cm, the concurrent use of medication, warm acupuncture, and TDP light therapy may accelerate suture degradation, thereby presenting a novel therapeutic strategy. In contrast, stubborn granulomas often require surgical removal. To minimize granulomas, it's important to place acupuncture thread sutures at the dermal‐subcutaneous junction and within the muscle, as the dermis is sensitive and reactive. Enhancing aseptic techniques, preventing biofilm, and evaluating patient factors before surgery can further decrease adverse reactions. Since there's no effective treatment for foreign body granulomas, prevention is essential.

## Author Contributions

All authors confirm their contributions to the paper as follows: S.H., X.X., K.S., Y.S., and C.J.: study conception and design. X.X.: data collection. All authors: interpretation of results. X.X.: draft manuscript preparation.

## Funding

This study was funded by the Zhejiang Province Health Talents Project (1S22213) and Zhejiang Provincial Hospital of Traditional Chinese Medicine Flying Eagle Talent Program 3.0 (2D02341).

## Ethics Statement

The authors confirm that the journal's ethical policies, as noted on the journal's author guidelines page, have been adhered to.

## Consent

Written consent was obtained from the patient.

## Conflicts of Interest

The authors declare no conflicts of interest.

## Data Availability

The data that support the findings of this study are available from the corresponding author upon reasonable request.
